# Activation of sorbitol pathway in metabolic syndrome and increased susceptibility to cataract in Wistar-Obese rats

**Published:** 2012-02-24

**Authors:** Paduru Yadagiri Reddy, Nappan Veettil Giridharan, Geereddy Bhanuprakash Reddy

**Affiliations:** 1 Biochemistry Division, National Institute of Nutrition, Hyderabad, India; 2 National Centre for Laboratory Animal Sciences, National Institute of Nutrition, Hyderabad, India

## Abstract

**Purpose:**

Obesity is a major public health problem worldwide, and of late, epidemiological studies indicate a preponderance of cataracts under obesity conditions. Although cataract is a multifactorial disorder and various biochemical mechanisms have been proposed, the influence of obesity on cataractogenesis has yet to be investigated. In such a scenario, a suitable animal model that develops cataract following the onset of obesity will be a welcome tool for biomedical research. Therefore, we investigated the molecular and biochemical basis for predisposition to cataract in the obese mutant rat models established in our institute because 15%–20% of these rats develop cataracts spontaneously as they reach 12–15 months of age.

**Methods:**

We analyzed the major biochemical pathways in the normal lenses of different age groups of our obese mutant rat strains, Wistar/Obese (WNIN/Ob) and WNIN/GR-Ob, the former with euglycemia and the latter with an additional impaired glucose tolerance trait. In addition, sorbitol levels were estimated in the cataractous lenses of the obese rats.

**Results:**

Except for the polyol pathway, all the principal pathways of the lens remained unaltered. Therefore, sorbitol levels were found to be high in the normal eye lenses of obese rats (WNIN/Ob and WNIN/GR-Ob) compared to their lean controls from three months of age onwards. Between WNIN/Ob and WNIN/GR-Ob, the levels of sorbitol were higher in the latter, suggesting a synergistic effect of impaired glucose tolerance along with obesity in the activation of the sorbitol pathway. Either way, an elevated sorbitol pathway seemed to be the predisposing factor responsible for cataract formation in these mutant rats.

**Conclusions:**

Activation of the sorbitol pathway indeed enhances the risk of cataract development in conditions such as metabolic syndrome. These rat models thus may be valuable tools for investigating obesity-associated cataract and for developing intervention strategies, based on these findings.

## Introduction

Epidemiological studies suggest the prevalence of overweight and obesity has reached epidemic proportions worldwide [1], and in the past 20 years, obesity rates have tripled in developing countries as well [2,3]. Several serious medical conditions are now associated with obesity, and it is no longer considered just a problem of overweight. Obesity is now aptly referred to as a metabolic syndrome and is associated with several degenerative diseases, including coronary heart disease, type 2 diabetes, hypertension, stroke, dyslipidemia, osteoarthritis, sleep apnea, fatty liver, and certain types of cancers. Eye problems are probably the latest addition to this list associated with obesity [4,5]. The ocular complications of obesity include diabetic retinopathy, high intraocular pressure, cataracts, macular degeneration, and exophthalmos [4,5].

Cataract is the leading cause of blindness worldwide and accounts for an estimated 16 million cases of blindness, with approximately half of all cases originating in Africa and Asia [6,7]. Recently, several large population-based studies have shown the association between obesity and cataract [5,8-12]. For example, the Physicians’ Health Study [9,12], the Nurses’ Health Study [10], and the Framingham Eye Study [11], as well as several cross-sectional studies [13-16], demonstrated a positive association between various measures of obesity and cataract. Cortical and posterior subcapsular cataracts have been most consistently associated with obesity [13,15-17]. In the Age-Related Eye Disease Study (AREDS), higher body mass index (BMI) and weight gain were found to have significant association with moderate cortical cataract [17].

There are three principal mechanisms by which the lens can be damaged, resulting in cataract (oxidative stress, osmotic effect, and non-enzymatic protein glycation [18]), and obesity might influence any or all of these physiologic processes [19,20]. However, to the best of our knowledge no experimental studies have explained the association between obesity and cataract and their plausible pathophysiological mechanism(s). Thus, there is an urgent need for mechanistic studies to understand cataract due to obesity. In such a scenario, the availability of an animal model that develops cataract following the onset of obesity (akin to obesity-associated cataract in patients) is of immense value.

At the National Center for Laboratory Animal Sciences of our institute, as early as 1997, a spontaneously developed obese rat was isolated from the existing Wistar (WNIN) stock of rats, and a colony of WNIN-Obese (WNIN/Ob) rats was established by selective breeding [21,22]. Subsequently, it bifurcated into another strain with impaired glucose tolerance (IGT)-WNIN/GR-Ob [21,23]. The phenotype and associated biochemical, histological, and pathophysiological characteristics of WNIN/Ob and WNIN/GR-Ob rats have been reported in detail elsewhere [21-24]. In essence, starting from 35 to 40 days of age, the WNIN/Ob and WNIN/GR-Ob phenotypes are different from their respective lean littermates, and the rats’ bodyweight increases progressively until the age of six to nine months. The average life span of these obese mutant rat strains was found to be 18–24 months as against 30–36 months for their parent WNIN strain. The animals show hyperinsulinemia, hypertriglyceridemia, and hypercholesterolemia. In addition, these rats also seem to develop a few degenerative conditions such as retinal degenerations as early as four months of age [25]. Further, our initial screening of these rat colonies showed that about 15% of WNIN/Ob and 20% of WNIN/GR-Ob rats develop cataracts spontaneously by the time they reach 12 months of age. However, the molecular basis for these cataracts was a dilemma, that, too, being shown in about only 15%–20% animals at best. We undertook the present study to explore and explain the observed enigma.

## Methods

### Materials

Anti-α-actin antibody, bovine serum albumin (BSA), 1-chloro-2,4-dinitro benzene (CDNB), 2,4-dinitro phenyl hydrazine (DNPH), diethylene triamine penta acetic acid (DEPTA), glucose-6-phosphate, glutathione reductase, DL-glyceraldehyde, glutathione (GSH), nicotinamide adenine dinucleotide (NAD), NADH, nicotinamide adenine dinucleotide phosphate (NADP), nicotinamide adenine dinucleotide phosphate (reduced) (NADPH), pyrogallol, D-sorbitol, sorbitol dehydrogenase, antirabbit immunoglobulin G (whole molecule) peroxidase, and Tris-HCl were purchased from Sigma Chemicals (St. Louis, MO). The Sephacryl S-300 HR was from Amersham Biosciences (Piscataway, NJ).

### Animals and tissue collection

All the procedures involving rats were performed in accordance with the Association for Research in Vision and Ophthalmology (ARVO) statement for the Use of Animals in Ophthalmic and Vision Research and were approved by the Institutional Animal Ethics Committee at the National Institute of Nutrition. Animals were kept in a 12-h light-dark cycle with ambient light intensity and temperature at the National Center for Laboratory Animal Science, National Institute of Nutrition. Three- to 12-month-old WNIN/Ob and WNIN/GR-Ob rats along with their respective lean littermate rats were fasted overnight and sacrificed by CO_2_ asphyxiation at the end of the dark cycle.

### Slit lamp examination and lens collection

Eyes were examined for lens opacity using a slit lamp biomicroscope (Kowa Portable; Kowa, Ltd., Tokyo, Japan) on dilated pupils. Only clear lenses without any opacity were used for analysis. Eyeballs of three- to 12-month-old animals were collected, and the lenses were dissected by the posterior approach. Briefly, a small incision was made on the posterior side of the eye with scissors. The lenses were collected by pressing with tweezers against the side of the eye opposite the incision and stored at −70 °C until further analysis.

### Protein solubility, crystallin distribution, protein cross-linking, and protein aggregation

A 10% homogenate of the lenses was prepared in homogenization buffer (25 mM Tris-Cl, pH 8.0 containing 0.5 mM ethylenediaminetetraacetic acid and 100 mM NaCl). The homogenate was centrifuged at 10,000× g for 30 min at 4 °C. The supernatant was referred to as the soluble fraction. Total and soluble protein content was estimated with the Lowry method, and the percentage of soluble protein was calculated. The soluble fraction was applied onto a 300× 7.8 mm TSK-3000 SW-XL size exclusion chromatography (SEC) column (Tosoh Co., Tokyo, Japan) using a Shimadzu high-performance liquid chromatography system [26]. The subunit profile and cross-linking of lens-soluble proteins were analyzed on 12% polyacrylamide gels in the presence of sodium dodecyl sulfate (SDS) under reducing conditions [27].

### Oxidative stress and antioxidant defense system

Lens lipid peroxidation was measured as thiobarbituric acid reacting substances (TBARS) and protein carbonyl content was determined based on their reactivity with 2,4-dinitrophenyl hydrazine according to reported methods [27-29]. In brief, malondialdehyde is the end product of lipid peroxidation of polyunsaturated fatty acids. Malondialdehyde was estimated by utilizing its reactivity with 2-thiobarbituric acid resulting pink coloured condensation product, a trimethine which was measured spectrophotometrically at 533 nm. The carbonyls react with 2,4-dinitrophenyl hydrazine to form protein hydrozones, which can be detected and quantified spectrophotometrically at 365 nm. The activities of antioxidant enzymes: superoxide dismutase (SOD), glutathione peroxidase (GPx), glutathione S-transferase (GST) and glucose-6-phosphate dehydrogenase (G6PD) were assayed spectrophotometrically according to the reported methods [27,29]. The assay of SOD is based on the ability of the enzyme to inhibit the auto-oxidation of pyrogallol. The rate of auto-oxidation of pyrogallol is measured following the change in absorbance at 420 nm. GPx catalyzes the oxidation of reduced glutathione by hydrogen peroxide or lipid peroxides to oxidized glutathione (GSSG). The rate of GSSG formation, a measure of enzyme activity, was monitored coupling with the glutathione reductase reaction where NADPH oxidation was followed at 340 nm. GST catalyzes the conjugation of toxic electrophilic compounds with glutathione. In GST assay formation of 1-chloro-2,4-dinitrobenzene-glutathione conjugate was monitored at 340 nm. G6PD catalyzes the oxidation of glucose-6-phosphate to 6- phosphogluconolactone in presence of NADP+. The rate of NADP reduction to NADPH was followed as a measure of enzyme activity of G6PD at 340 nm.

### Non-enzymatic glycation

The extent of glycation was measured by monitoring advanced glycation end-product (AGE) related non-tryptophan fluorescence. AGE fluorescence measurements were performed using a Jasco spectrofluorometer (FP-6500; Tokyo, Japan) in soluble protein (0.15 mg/ml protein in 50 mM sodium phosphate buffer, pH 7.2) [28]. Fluorescence spectra were obtained from 400 to 500 nm with excitation at 370 nm.

### Polyol pathway

The status of the polyol pathway in the eye lenses of WNIN/Ob and WNIN/GR-Ob rats was assessed by analyzing the activity of aldose reductase (ALR2) and sorbitol dehydrogenase (SDH) and sorbitol levels in the lens. The activities of ALR2 and SDH were assayed spectrophotometrically according to the reported methods [27,29]. ALR2 catalyzes the NADPH linked reduction of glyceraldehyde and change in the absorbance at 340 nm due to NADPH oxidation was monitored. SDH catalyses the NAD linked oxidation of sorbitol to fructose or reduction of fructose to sorbitol. SDH activity was measured by the decrease in absorbance at 340 nm due to the oxidation of NADH using fructose as the substrate.. Sorbitol was extracted by homogenizing the lens in nine volumes of 0.8 M perchloric acid. The homogenate was centrifuged at 5,000× g at 4 °C for 10 min, and the pH of the supernatant was adjusted to 3.5 with 0.5 M potassium carbonate. The sorbitol content of the supernatant was measured with the fluorometric method as described previously [30] using a fluorometer (Jasco-FP-6500, Tokyo, Japan). One ml reaction mixture, consisted of 50 μmol glycine buffer, pH 9.4, 2 μmol magnesium chloride, 0.2 μmol nicotinamide adenine dinucleotide (NAD) and protein-free supernatant, was incubated for 5 min at 37 °C, and the reaction was initiated by the addition of 0.6 U of SDH. The relative fluorescence due to NADH formation was measured in a fluorometer with an excitation wavelength at 360 nm and an emission wavelength of 452 nm. Sorbitol standards, ranging from 0.2 to 9.0 μg/ml, were analyzed the same way to generate a standard curve. Sorbitol was also estimated in a few cataractous lenses collected from the obese rats.

### Immunodetection of ALR2 in the soluble fraction of lens

Lens-soluble proteins were resolved under reducing conditions on 12% SDS–polyacrylamide gel electrophoresis (PAGE), proteins were transferred onto a nitrocellulose membrane (NC), and NC was blocked with 5% skimmed milk powder. The NC membrane was incubated with an affinity purified polyclonal antisera of ALR2 (1:2,000 dilution) later with horseradish peroxidase–conjugated goat antirabbit antibody (1:2,500). Subsequently, detection was performed with diaminobenzidine in the presence of hydrogen peroxide.

### Statistical analysis

Descriptive statistics were calculated for all the study variables, and a *t* test was used to compare the mean values of different parameters among groups for given age/strain. Mean values were also compared among the time points for given group/strain using ANOVA with a post hoc Fisher’s least significant difference test. Level of significance was considered as 0.05.

## Results and Discussion

### Protein content

Insolubilization and aggregation of soluble lens proteins is the major biochemical alteration leading to cataractogenesis. Therefore, we determined the total and soluble protein content in the lens of the WNIN/Ob and WNIN/GR-Ob rats at different ages. Although irrespective of phenotypes there was a decrease in soluble protein content with age, there was no difference in protein (total, soluble, and insoluble) content of the WNIN/Ob rats as compared to lean rats of the corresponding age (Table 1). Similar results were observed with WNIN/GR-Ob rats (data not shown).

**Table 1:** Protein content of eye lens.

**Age (months)**	**Group**	**Total protein (mg/g lens)**	**Soluble protein (mg/g lens)**	**Soluble protein (%)**
3	Lean	502.8±15.81	389.2±19.57	77.54±2.999
	Obese	497.4±15.05	381.7±11.99	77.17±2.517
6	Lean	573.0±12.64	391.7±11.44	68.34±1.319
	Obese	564.5±16.73	379.8±14.01	67.36±1.507
12	Lean	612.4±15.04	369.1±10.09	60.33±1.208
	Obese	619.6±28.71	356.6±8.12	58.19±1.958

The total and soluble protein in the lens of WNIN/Ob rats of different age was estimated by Lowry’s method and the percentage soluble protein content was derived from the estimated values. Data are mean±SE (n=6).

### Crystallin distribution and protein cross-links

**Figure 1:**
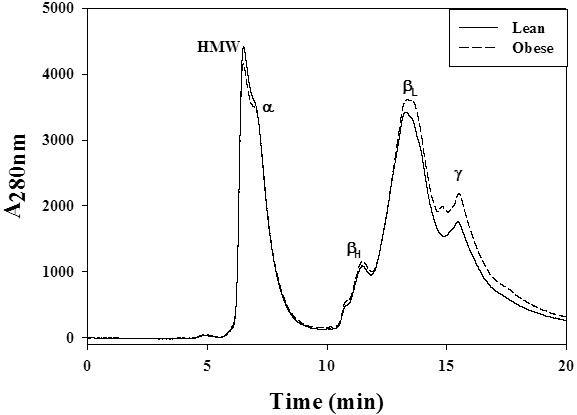
Profile of crystallin distribution in WNIN/Ob rat lens. Representative distribution profile of eye lens crystallins in soluble fraction of lean and WNIN/Ob rats. Soluble protein (20 μl, 1 mg/ml in equilibration buffer) was loaded on TSK-G3000 SWXL gel filtration column and protein peaks were detected at 280 nm with a flow rate of 1 ml/min. Peaks representing alpha (α-), betaH (βH-), betaL (βL-), and gamma (γ-) crystallin, and high molecular weight (HMW) fractions are indicated at their respective positions.

**Figure 2:**
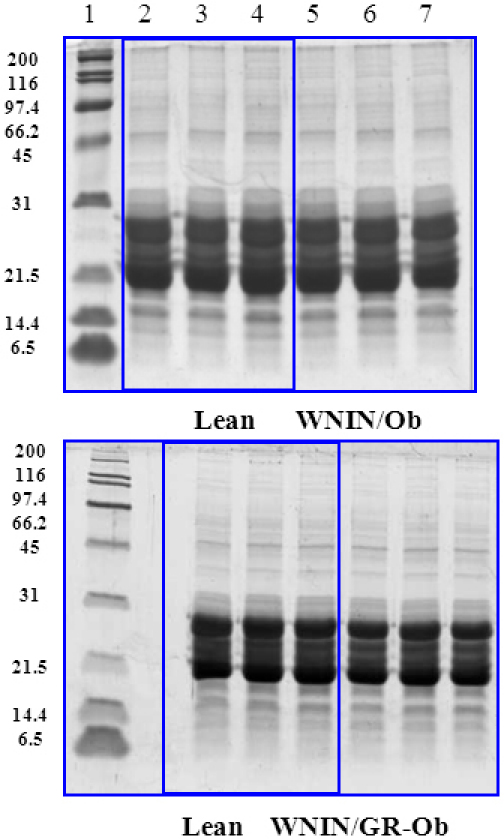
Subunit profile of lens proteins of WNIN/Ob and WNIN/GR-Ob rat. Representative subunit profile and protein cross-linking of the soluble fraction of lens of WNIN/Ob (top panel) and WNIN/GR-Ob rats (bottom panel) at different ages. Soluble lens protein was resolved on a 12% polyacrylamide gel under reducing conditions. Lane 1: molecular weight markers, lane 2: lean (3-months), lane 3: lean (6-months), lane 4: lean (12-months), lane 5: obese (3-months), lane 6: obese (6-months) and lane 7: obese (12-months).

To understand the possible alterations in crystallin distribution and subunit profile due to obesity, the soluble protein fraction was analyzed with SEC and SDS–PAGE. The lens-soluble fraction was clearly resolved into high molecular weight aggregate-, αL-, β-, and γ-crystallin peaks on a SEC column, and there was no difference in the percentage distribution of different crystalline peaks between WNIN/Ob and its respective lean animals from three to 12 months (Figure 1). Similar results were observed with WNIN/GR-Ob rats (data not shown). Similarly, the subunit profile of lens proteins and cross-linking was also not different in WNIN/Ob or WNIN/GR-Ob rats compared to their respective lean animals as assessed with SDS–PAGE (Figure 2).

### Oxidative stress and the antioxidant system

Increased oxidative stress has been implicated in the development of various types of cataract [31-34]. Therefore, we assessed oxidative stress by measuring lipid peroxidation, protein carbonyls, and some of the antioxidant enzymes in the lenses of WNIN/Ob and WNIN/GR-Ob rats. The TBARS levels, an indication of lipid peroxidation, were not different in the WNIN/Ob or WNIN/GR-Ob lenses compared with their respective lean controls from three to 12 months (data not shown). Usually, increased lipid peroxidation is observed in hypercholesterolemia and hyperlipidemia. Although the obese rats were hypercholesterolemic and hyperlipidemic, we did not observe any change in the lipid peroxidation of the lenses between the lean and obese rats suggesting that the lens might have a robust mechanism to preserve and maintain the fatty acids profile even under altered metabolic conditions. Because we observed a significant increase in lipid peroxidation (TBARS) in other tissues such as the livers, hearts, and kidneys of the WNIN/Ob and WNIN/GR-Ob rats compared with their respective lean animals (data not shown). Further, there were no significant differences in the fatty acid composition of the lenses between the lean and WNIN-Ob rats (data not shown), which indicates that indeed the eye lens has a robust mechanism for preserving and maintaining the fatty acids profile under altered metabolic conditions.

**Figure 3:**
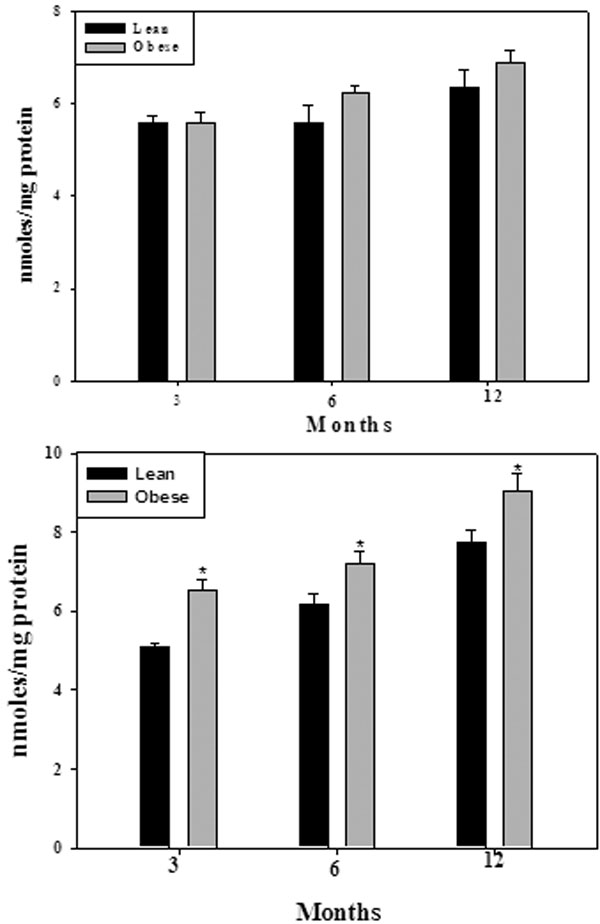
Protein carbonyl content in soluble protein fraction of WNIN/Ob (top panel) and WNIN/GR-Ob rats (bottom panel) at different ages. Data are mean±SE (n=6). The asterisk (*) above the bars denotes that data are significantly different between lean and obese rats at the respective age.

However, we found the protein carbonyl content, a measure of oxidative damage to proteins, higher in the WNIN/Ob and WNIN/GR-Ob rat lenses compared with their respective lean controls suggesting enhanced protein oxidation in the obese rat lens (Figure 3). The increase in protein oxidation due to obesity was more prominent in the WNIN/GR-Ob rat lenses compared to the WNIN/Ob rat lenses (Figure 3) indicating the added effect of IGT. Similarly, there was an increase, though statistically not significant, specifically SOD, GPx, G6PD, and GST activities in the lenses of WNIN/Ob and WNIN/GR-Ob animals compared with their respective lean animals at three, six, and 12 months, thereby substantiating the increased oxidative stress in the eye lens due to obesity (Table 2).

**Table 2:** Activity of antioxidant enzymes.

**Age (months)**	**Group**	**SOD**	**GPx**	**G6PD**	**GST**
3	Lean	28.42±2.675	22.83±1.467	7.38±0.717	32.88±1.826
	Obese	30.42±2.383	24.34±1.131	8.19±0.681	35.07±3.433
6	Lean	31.20±2.028	21.04±1.581	7.37±0.455^a^	27.20±1.382
	Obese	33.10±2.303	23.63±2.207	9.39±0.599^b^	26.13±2.064
12	Lean	32.1±2.972	21.87±1.665	6.19±0.392^a^	32.77±2.227
	Obese	38.08±2.519	25.92±0.912	7.39±0.249^b^	34.22±1.561

Activity of superoxide dismutase (SOD), glutathione peroxidase (GPx), glucose-6-phosphate dehydrogenase (G6PD), and glutathione-S-transferase (GST) in the eye lens of WNIN/Ob rats of different age. Data are mean±SE (n=6). Different superscripts denote that data are significantly different between lean and obese rats at the respective age. Specific activity was expressed as Units/100 mg protein (SOD) or μmoles NADPH oxidized/h/100mg protein (GPx) or μmoles NADP reduced/h/100 mg protein (G6PD) or μmoles GSH-CDNB conjugate formed/h/100 mg protein (GST).

### Non-enzymatic glycation

Non-enzymatic glycation of lens protein has been considered a major factor responsible for age-related and diabetic cataracts [35,36], which alters protein conformation and stability, induces protein aggregation and cross-linking, and leads to protein insolubilization [37-39]. Hence, the degree of glycation in the soluble and insoluble protein fraction of the WNIN/Ob and WNIN/GR-Ob animals was measured by monitoring AGE-related non-tryptophan fluorescence. However, there was no difference in AGE-related non-tryptophan fluorescence between WNIN/Ob and WNIN/GR-Ob and the respective lean rats in all the age groups studied (data not shown). The lack of change in the subunit profile and cross-linking of lens proteins in WNIN/Ob or WNIN/GR-Ob rats compared to their respective lean animals (Figure 2) further corroborate these observations.

### Polyol pathway

**Figure 4:**
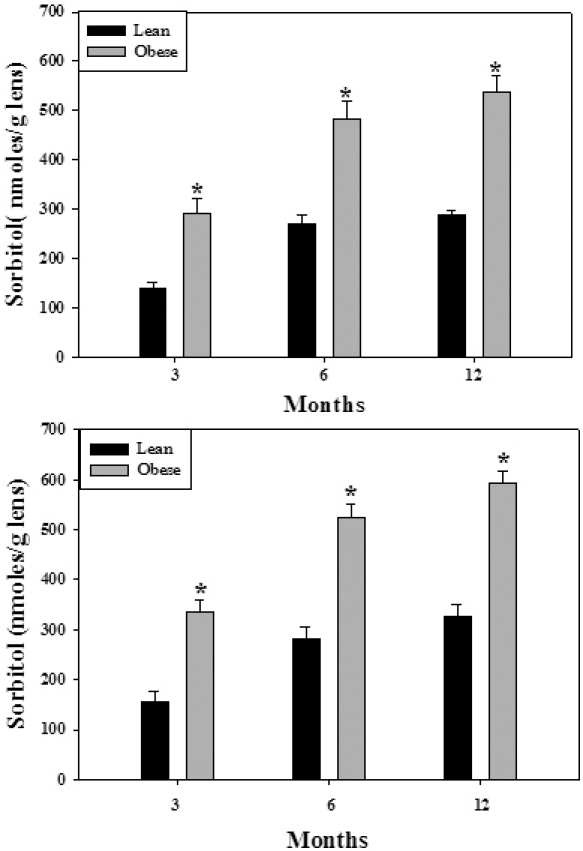
Sorbitol levels in the eye lens of WNIN/Ob (top panel) and WNIN/GR-Ob rats (bottom panel) at different ages. Data are mean±SE (n=6). The asterisk (*) above the bars denotes that data are significantly different between lean and obese rats at the respective age.

**Figure 5:**
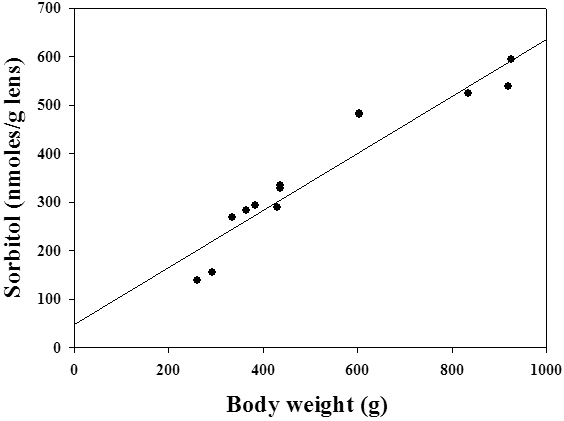
Correlation between body weight and lens sorbitol of WNIN/Ob and WNIN/GR-Ob rats. Correlation (r=0.958) between eye lens sorbitol levels and body weight of WNIN/Ob and WNIN/GR-Ob rats and their corresponding lean controls at different ages was found to be highly significant (p<0.001).

Among the many biochemical pathways associated with cataract, the polyol pathway has been extensively studied, particularly in the diabetic cataract [29,34,40,41]. Hence, we investigated the status of the polyol pathway in WNIN/Ob and WNIN/GR-Ob rat lenses. The specific activity of ALR2, the rate-limiting enzyme of the polyol pathway, was moderately but consistently higher in the lenses of WNIN/Ob and WNIN/GR-Ob animals from three months of age onwards compared to the respective lean animals (Table 3). However, the activity of SDH, the second enzyme of the polyol pathway, was not altered significantly in obese rats (data not shown). Yet there was a remarkable increase in sorbitol, the product of ALR2, in the lenses of the WNIN/Ob and WNIN/GR-Ob rats when compared to respective lean animals from three months of age onwards (Figure 4). The increase in sorbitol levels was greater in the WNIN/GR-Ob rats compared to the WNIN/Ob rats for the corresponding age groups, and the increase was higher in older animals (Figure 4). This further indicates a synergistic effect of IGT along with obesity on the activation of the sorbitol pathway. Increased levels of sorbitol were consistent with the increased bodyweight as sorbitol levels were highly correlated with bodyweight with a correlation coefficient of 0.958 (p<0.001; Figure 5). This suggests that BMI (overweight or obesity) has a direct influence on sorbitol levels.

**Table 3:** Aldose reductase activity.

** **	**WNIN/Ob**		**WNIN/GR-Ob**	
**Age (months)**	**Lean**	**Obese**	**Lean**	**Obese**
3	36.56±1.869	40.08±2.524	37.64±1.313	39.44±2.080
6	37.89±1.639	41.33±1.818	40.40±1.269	43.84±2.383
12	39.68±1.459	43.88±1.855	41.04±1.894	45.16±1.414

Aldose reductase (ALR2) activity in WNIN/Ob and WNIN/GR-Ob rat lens of different age. Data are mean±SE (n=6). ALR2 activity was expressed as μmoles NADPH oxidized/h/100 mg protein.

**Figure 6:**
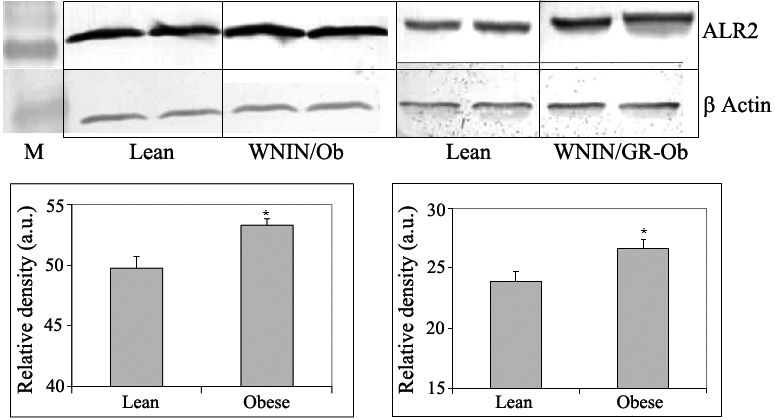
Immunodetection of aldose reductase in the eye lens of WNIN/Ob and WNIN/GR-Ob rats. Western blot of aldose reductase in the eye lens of WNIN/Ob (left panel) and WNIN/GR-Ob rats (right panel) at 12-months age. Data are mean±SE (n=6). The asterisk (*) above the bars denotes that data are significantly different between lean and obese rats.

For a moderate increase in ALR2 activity, the increase in sorbitol levels was substantial in the WNIN/Ob lenses and more so in the WNIN/GR-Ob rat lenses. Therefore, we then determined the expression of ALR2 in the lens with immunodetection using ALR2-specific polyclonal antibodies [42]. The data indicate ALR2 expression was higher in the lenses of WNIN/Ob and WNIN/GR-Ob rats compared to lean animals (Figure 6). The increase in transcript levels (analyzed with real-time polymerase chain reaction) also supports the western data (data not shown).

Apart from the major biochemical pathways, chaperone-like activity (CLA) of α-crystallin was also assessed, as the CLA of α-crystallin has been shown to play a critical role in maintaining lens transparency, and loss of CLA of α-crystallin was observed in many types of cataract [43]. However, there was no significant difference in the CLA of α-crystallin in the three- to 12-month-old WNIN/Ob and WNIN/GR-Ob eye lenses compared to their respective age-matched lean controls (data not shown).

**Figure 7:**
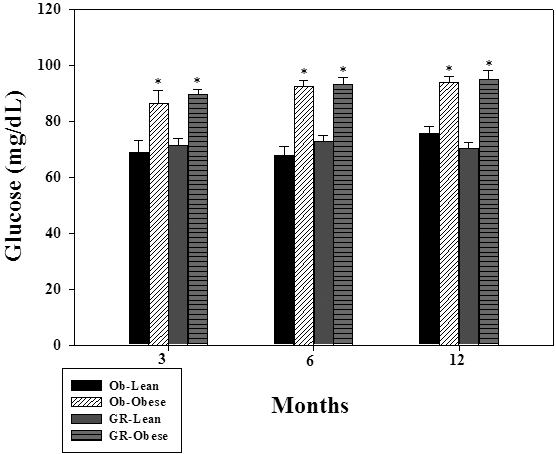
Plasma glucose levels of WNIN/Ob and WNIN/GR-Ob rats at different ages. Data are mean±SE (n=6). The asterisk (*) above the bars denotes that data are significantly different between lean and obese rats at the respective age.

These results indicate that under normal conditions all principal lens pathways except the polyol pathway remained unaltered in these mutant rats. The higher levels of sorbitol in the lenses of the WNIN/Ob and WNIN/GR-Ob rats could be attributed to increased specific activity as well as increased expression of ALR2. Further, there were moderately higher plasma glucose levels in the WNIN/Ob and WNIN/GR-Ob rats (Figure 7). Probably these three factors (chronic high glucose, increased activity, and expression of ALR2) together might have contributed to substantially high sorbitol levels in the lenses of the WNIN/Ob and WNIN/GR-Ob rats. Studies indicate a metabolic connection between the polyol pathway and oxidative stress [29,34,44]. Therefore, activation of the polyol pathway may lead to increased oxidative stress as indicated by higher protein carbonyls and altered activities of antioxidant enzymes in the eye lenses of the WNIN/Ob rats and more so in the WNIN/GR-Ob rats. These results suggest that increased sorbitol might be a predisposing factor for the higher incidence of cataract in WNIN/Ob and WNIN/GR-Ob rats.

A previous study reported increased sorbitol pathway activity in the sciatic nerve of the leptin-deficient *ob/ob* mouse with mild hyperglycemia that developed peripheral diabetic neuropathy (PDN) at 11 weeks [45]. Interestingly, administration of fidarestat, an ALR2 inhibitor, to *ob/ob* mice for six weeks was associated with alleviation of PDN pathogenic features [45], suggesting the involvement of sorbitol in PDN associated with obesity.

Another study also demonstrated that prediabetic neuropathy in mice fed a high-fat diet could be alleviated by a dietary intervention or a combination of a dietary intervention and pharmacological treatment with an ALR2 inhibitor [46], corroborating the role of sorbitol in the development of obesity associated complications. Increased accumulation of sorbitol in the retina has been implicated in the pathogenesis of diabetic retinopathy [30]. Rajala et al. examined insulin receptor (IR) signaling in the sorbitol-treated retina under ex vivo conditions and showed that sorbitol activates the IR and IGF-1R tyrosine kinases, which results in activation of the receptor’s direct downstream targets [47]. Recently we reported a threefold increase in sorbitol levels in the eye lens (without cataract) in the neonatal streptozotocin rat model with prolonged IGT and insulin resistance [48]. All these studies support a preponderance of sorbitol accumulation and cataract in WNIN/GR-Ob rats (with IGT) over WNIN/Ob rats.

However, it is an enigma why only 15%–20% obese animals in the WNIN/Ob and WNIN/GR-Ob colony develop cataracts if accumulation of sorbitol is the cause of cataract formation in these strains. Sorbitol levels could reach or cross a threshold level to develop cataracts. To substantiate this assumption, we estimated sorbitol levels in the cataractous lenses of WNIN/Ob and WNIN/GR-Ob rats at random. Sorbitol levels in the cataractous lens of four- to six-month-old WNIN/Ob and WNIN/GR-Ob rats were between 650 and 750 nmoles/g lens (mean 680 nmoles; n=10), just above the levels (650 nmoles) as seen in 12-month-old WNIN/Ob and WNIN/GR-Ob rats (Figure 2). This indicates that sorbitol levels above 650 nmoles may result in cataracts in these animals. Any factor (such as fluctuations in glucose levels, ALR2 activity, or ALR2 expression) that abets or incites reaching this threshold level for sorbitol could precipitate cataract formation. Increased susceptibility of WNIN/Ob and WNIN/GR-Ob rats to galactose- and streptozotocin-induced cataract compared to lean rats (P.Y.R., N.V.G., and G.B.R. unpublished data) further supports early onset and higher incidence of cataracts in WNIN/Ob and WNIN/GR-Ob rats. Although factors other than the increased intracellular glucose concentrations that cause sorbitol accumulation need to be identified, these findings are in line with similar observations made previously in models of nondiabetic conditions and aging [49,50]. However, since the nature of the genetic lesion in these rats at present is unknown (work in progress), it is not possible to directly attribute any pathological process to this metabolic phenotype. It could be even independently related to the genotype.

In summary, the results indicate that activation of the sorbitol pathway appears to enhance the risk of cataract development in WNIN-obese rats. These rat models of metabolic syndrome may thus be valuable tools for investigating obesity-associated cataract and for developing intervention strategies based on these observations. The data presented here also imply that there might be a connection between increased sorbitol levels and complications associated with obesity (or metabolic syndrome), which needs to be investigated further.
